# The *Mycobacterium tuberculosis* PE Proteins Rv0285 and Rv1386 Modulate Innate Immunity and Mediate Bacillary Survival in Macrophages

**DOI:** 10.1371/journal.pone.0051686

**Published:** 2012-12-17

**Authors:** Bhavana Mishra Tiwari, Nisha Kannan, Lakshmi Vemu, Tirumalai R. Raghunand

**Affiliations:** 1 CSIR - Centre for Cellular and Molecular Biology, Hyderabad, India; 2 Nizam’s Institute of Medical Sciences, Hyderabad, India; University of Maryland, United States of America

## Abstract

The unique PE/PPE multigene family of proteins occupies almost 10% of the coding sequence of *Mycobacterium tuberculosis* (*M.tb*), the causative agent of human tuberculosis. Although some members of this family have been shown to be involved in pathways essential to *M.tb* pathogenesis, their precise physiological functions remain largely undefined. Here, we investigate the roles of the conserved members of the ‘PE only’ subfamily Rv0285 (PE5) and Rv1386 (PE15) in mediating host-pathogen interactions. Recombinant *Mycobacterium smegmatis* strains expressing PE5 and PE15 showed enhanced survival *vs* controls in J774.1 and THP-1 macrophages - this increase in viable counts was correlated with a reduction in transcript levels of inducible nitric oxide synthase. An up-regulation of anti- and down-regulation of pro-inflammatory cytokine levels was also observed in infected macrophages implying an immuno-modulatory function for these proteins. Induction of IL-10 production upon infection of THP-1 macrophages was associated with increased phosphorylation of the MAP Kinases p38 and ERK1/2, which was abolished in the presence of the pharmacological inhibitors SB203580 and PD98059. The *PE5-PPE4* and *PE15-PPE20* gene pairs were observed to be co-operonic in *M.tb*, hinting at an additional level of complexity in the functioning of these proteins. We conclude that *M.tb* exploits the PE proteins to evade the host immune response by altering the Th1 and Th2 type balance thereby favouring *in vivo* bacillary survival.

## Introduction

The extraordinary success of *M.tb*, the etiologic agent of human tuberculosis (TB), has been attributed to its ability to modulate host immune responses facilitating its long term persistence [1]. The identification and characterisation of bacillary (virulence) factors involved in evasion, and their interplay with host defence components during infection is vital to understanding the pathogenic mechanisms of *M.tb*. In this context, the multigenic PE/PPE proteins (named after the conserved Proline-Glutamate and Proline-Proline-Glutamate residues at their N-termini), which comprise about 10% of the coding potential of the *M.tb* genome have emerged as a central figure, as several members of this family have been implicated in host immune evasion [2]. The PE sub-group consist of 34 proteins of the ‘PE only’ class characterised by a relatively conserved N-terminus of ∼110 aa, and 67 PE_PGRS (polymorphic GC rich repetitive sequences) proteins with a C-terminal region comprised of glycine-rich repeat regions ranging from ∼100 to over 500 aa in length. Members of this family are present only in pathogenic mycobacteria indicative of their importance in disease pathogenesis [3]. Of the 69 PPE proteins, 22 are present in predicted operonic clusters exclusively containing PE/PPE genes [2], [4]. 14 of these are gene pairs with a 5′*PE*-*PPE* 3′ orientation with no more than 100 bp separation between the genes, suggesting that these may be co-transcribed and also be functionally linked [5]. Comparative genomics suggests that the evolution and expansion of the PE/PPE family is closely linked with ESAT-6 (*esx*) like genes - the 5 *esx* clusters (*esx1-5*) scattered across the *M.tb* genome, encode components of Type VII secretion systems. PE35 (Rv3872) is believed to be the first PE insertion into the *esx*-1 gene cluster followed by duplication and divergence leading to the expansion of this gene family [3].

Several important functions have been attributed to members of the PE sub-group. The PE_PGRS proteins localize to the cell wall and are available on the mycobacterial cell surface [6], [7], [8]. Consistent with this observation, the PE domain of PE_PGRS33 has been shown to contain the information necessary for translocation and cell wall localisation [9], [10]. Several members of this family have been shown to be immunogenic [11], [12], [13] and it has also been suggested that they may provide a mechanism for generating antigenic diversity in mycobacteria [7], [14]. Ectopic expression of PE_PGRS33 was observed to trigger apoptosis in Jurkat T cells indicating its role in *M.tb* virulence [15]. Also, execution of macrophage apoptosis by this protein was seen to be mediated by toll like receptor 2 (TLR2) dependent release of TNFα, and deletions within the PGRS domain attenuated its TNFα-inducing ability. This suggested that variations in the polymorphic repeats of the PGRS domain modulate the innate immune response [16]. Two *Mycobacterium marinum* homologues of the PE_PGRS family were found to be specifically expressed in granulomas [17]. Evidence also exists for their variable expression in *M.tb* infected macrophages and in the mouse model of *M.tb* infection [18]. Gene expression profiling of *M.tb* exposed to 15 unique conditions relevant to its pathogenesis revealed that the expression of the PE/PPE genes is controlled by a variety of independent mechanisms. This differential expression could potentially provide a dynamic antigenic profile during the course of changing microenvironments within the host [19]. Although, much information exists in the literature on the PE_PGRS sub-group, the ‘PE only’ class of proteins are poorly characterised with regard to their functional role in *M.tb* pathogenesis, and their relevance to the biology of *M.tb*.

To elucidate their significance *vis-a-vis M.tb* physiology, we chose to functionally characterise Rv0285 (PE5) and Rv1386 (PE15), two prototypical members of this sub-family. Infection of macrophages with recombinant *Mycobacterium smegmatis* (*M.smegmatis*) strains expressing the two proteins, led to the identification of their probable roles in intracellular persistence. The increased survival of these strains was accompanied by an alteration in the balance of pro- and anti-inflammatory cytokine levels with concurrent induction of the MAP kinase pathway. This study represents the first demonstration of an independent immuno-modulatory function for the ‘PE only’ sub-family of proteins and suggests that they could potentially play a pivotal role in the evasion of host immune response by *M.tb*.

## Materials and Methods

### Bacterial Strains, Media and Growth Conditions

The mycobacterial strains *M.smegmatis* mc^2^6 and *M.tb* H37Ra were cultured in Middlebrook 7H9 broth and 7H10 agar (Difco) containing albumin dextrose complex (5 g BSA, 2 g glucose and 0.85 g NaCl/L), 0.5% (v/v) glycerol and 0.05% Tween 80. *E.coli* strains were cultured in Luria Bertani media. Both *E. coli* and mycobacteria were grown at 37°C with shaking. Antibiotics were added when necessary: ampicillin (200 µg/mL), kanamycin (50 µg/mL for *E. coli* and 15 µg/mL for mycobacteria). All recombinant *M.smegmatis* strains were cultured in the presence of 15 µg/mL of kanamycin.

### DNA Techniques

Restriction enzymes and T4 DNA ligase were purchased from New England Biolabs (NEB), and Taq polymerase was purchased from Invitrogen. Protocols for DNA manipulations, including plasmid DNA preparation, restriction endonuclease digestion, agarose gel electrophoresis, isolation and ligation of DNA fragments, and *E. coli* transformation were performed as described [20]. PCR amplifications were carried out according to the manufacturer’s specifications. Each of the 30 cycles was carried out at 95°C for 30 s, 60°C for 30 s and 72°C for 1 min, followed by a final extension cycle at 72°C for 10 min. DNA fragments used for cloning reactions were purified by using the Qiagen gel extraction kit according to the manufacturer’s specifications. *M.smegmatis* was transformed by electroporation.

### *In silico* Analyses

Multiple Sequence Alignments were performed using the ClustalW2 algorithm [21] and the output files were imported into Boxshade 3.21 (http://www.ch embnet.org) to generate the formatted alignments. All *M.tb* sequences were obtained from the Tuberculist database (http://tuberculist.epfl.ch/). The TM-pred [22] and Kyte & Doolittle [23] algorithms were used to identify trans-membrane domains and regions of hydrophobicity respectively. Phylogenetic analysis was carried out using the Molecular Evolutionary Genetics Analysis (MEGA ver5) package [24] to generate a bootstrapped Unweighted Pair Group Method with Arithmetic Mean (UPGMA) tree from >500 replicates.

### Sub-cellular Localisation and Proteinase K Sensitivity Assay

To determine their localisation, C-terminal myc fusions of *M.tb PE5* and *PE15* were generated by cloning the ORFs between the *Bam*HI and *Eco*RI sites of pJEX55 [25] and the recombinant plasmids transformed into *M.smegmatis*. Recombinant *M.smegmatis* strains expressing c-myc tagged PE5 and PE15 were harvested at the logarithmic phase of growth, washed and resuspended in PBS. Each sample was divided into two identical aliquots and incubated at 37°C for 30 min with or without 100 µg/mL of Proteinase K (Sigma). The reaction was stopped by the addition of 2 mM EGTA and sub-cellular fractions of these samples were isolated as described [9]. Individual fractions were separated by SDS PAGE and the fusion proteins were detected by immunoblotting with the anti c-myc monoclonal antibody (mAb) 9E10 (sc40, Santa Cruz)**.**

### Expression of PE5 and PE15 in *M.smegmatis*

To functionally characterise *M.tb* PE5 and PE15, their ORFs were amplified from *M.tb* H37Rv genomic DNA using gene specific primers (Table S1), cloned between the *Bam*HI and *Eco*RI sites of pMV261 [26] and transformed into *M.smegmatis*.

### *In vitro* Growth Kinetics

To examine their growth patterns, recombinant *M.smegmatis* strains were grown until late exponential phase, diluted to an optical density (OD, A_600_) of 0.2 and cultured in Middlebrook 7H9 containing 15 µg/mL kanamycin. Growth curves were generated by OD and colony forming unit (CFU) measurements and plotted against time. At each designated time point, cultures were harvested for RNA extraction and gene expression analyses. All growth and CFU enumeration assays were performed in the presence of 15 µg/mL kanamycin.

### Macrophage Infection

J774.1 and THP-1 macrophages were cultured at 37°C in 5% CO_2_ in RPMI 1640 medium supplemented with 10% (v/v) Fetal bovine serum, 2 g/L sodium bicarbonate and antibiotics (60 µg/mL penicillin G sodium, 50 µg/mL streptomycin sulphate, and 30 µg/mL gentamycin sulphate). J774.1 cells were seeded in 6 well plates at a density of 0.5×10^5^ cells/well and used for infection 24h later. THP-1 monocytes were seeded at a density of 2×10^6^/well, differentiated with 5 ng/mL phorbol-12-myristate-13-acetate (PMA) for 24 h, and infected 72 h later. Exponentially growing bacteria cultured in the presence of 15 µg/mL kanamycin were pelleted, washed and resuspended in RPMI medium (without antibiotics) to an OD of 1.0. Single cell suspensions of recombinant *M.smegmatis* strains were obtained by passing cultures 5–6 times through 26 ½ gauge needles. Bacillary viability was assessed at each step by performing CFU counts. Equal numbers of each strain were used to perform infections (input counts) at a multiplicity of infection (MOI) of 1∶100, chosen based on pilot infections with multiple MOIs that we performed with the cell lines used. For activation, cells were stimulated overnight with 20 ng/mL Interferon-γ (IFN-γ), followed by a 3 h treatment with 200 ng/mL of lipopolysaccharide (LPS). After incubation with bacteria for 2 h, cells were washed with Phosphate Buffered Saline (PBS) and post-infection CFU counts determined by lysis of infected cells (T_0_ counts). Following this, complete RPMI containing gentamycin was added to kill extracellular bacteria. CFU counts were determined at the designated time points by lysing infected cells with 0.1% Triton X-100 followed by dilution plating on Middlebrook 7H10 agar. In each experiment, a sample of macrophages infected with *M.smegmatis* expressing the empty vector was included as the control.

### Real-time PCR Analysis

To determine expression profiles of *PE5* and *PE15* in recombinant strains of *M.smegmatis* expressing pMV261PE5 and pMV261PE15 as a function of growth, cells were harvested at the 4, 6, 8, 12, 24, 48 and 72 h time points and total RNA isolated from each culture using TRIzol reagent (Invitrogen) as per the manufacturer’s protocol. Following treatment with RNAse free DNAse I, cDNA synthesis was performed using the iScript cDNA synthesis kit (Bio-Rad) and subsequently used as a template for SYBR green based PCR amplification using *PE5* and *PE15* specific primers (Table S1) to generate 200 bp amplicons. Gene specific transcript levels were normalised to the *M.smegmatis sigA* transcript in each sample. The relative fold change in transcript levels at each time point was calculated with respect to the levels at 4 h. To quantify cytokine and *iNOS* transcript levels, total RNA was isolated from infected macrophages using TRIzol reagent. Following treatment with RNAse free DNAse I, cDNA synthesis was performed using the iScript cDNA synthesis kit (Bio-Rad) and subsequently used as a template for SYBR green based PCR amplification using gene specific primers (Table S1) designed to generate 200 bp amplicons. The levels of each mRNA was normalised to the transcript levels of GAPDH and β-actin. Relative fold change was calculated with reference to macrophages infected with *M.smegmatis* expressing the empty vector.

### Cytokine Assays

Levels of IL-10 and IL-12 p70 in the culture supernatants of infected THP-1 macrophages were quantitated by a two-site sandwich EIA (Becton Dickinson OptEIA) as per the manufacturer’s protocol.

### Western Blotting

To determine total and phosphorylated p38 and ERK1/2 mitogen activated protein kinase (MAPK) levels, infected THP-1 cells were lysed in RIPA lysis buffer. Equal protein amounts from each cell lysate were subjected to SDS-PAGE and transferred onto PVDF membranes. Each blot was sequentially probed with antibodies specific to phosphorylated or total p38 MAPK and ERK1/2 MAPK (Cell Signaling Technology) and β-actin. Proteins were detected using Enhanced Chemiluminescence (Thermo-Fisher). For the inhibition studies, THP-1 cells were treated with 5 µg/mL SB203580 and 25 µM PD98059 (Sigma), 2 h prior to infection. DMSO was used as a vehicle control. For quantitation, the intensity of all bands was densitometrically measured using ImageJ and normalised to β-actin. The normalised levels of phosphorylated p38 and ERK1/2 in control samples (pMV261) were assigned a value of 1, and the fold change in the levels of these proteins in the test samples (PE5, PE15) were determined with respect to the control.

### Co-transcriptional Analysis

To examine the transcriptional status of the *PE5-PPE4* and *PE15-PPE20* gene pairs, total RNA was isolated from exponentially growing *M.tb* H37Ra as described above. DNAse I treated RNA was used as a template for cDNA synthesis using the primers *PPE4* R1 (4R1) and *PPE20* R1 (20R1) (Table S1) respectively. The generated cDNA was used for PCR amplification using combinations of gene specific primers. Appropriate negative controls (- RT) were included in the analysis.

### Statistics

For all experiments, the student’s t-test was conducted to determine statistical significance between two groups, when required.

## Results

### PE5 (Rv0285) and PE15 (Rv1386) are Evolutionarily Related and Conserved throughout the *M.tb* Complex

**Figure 1:**
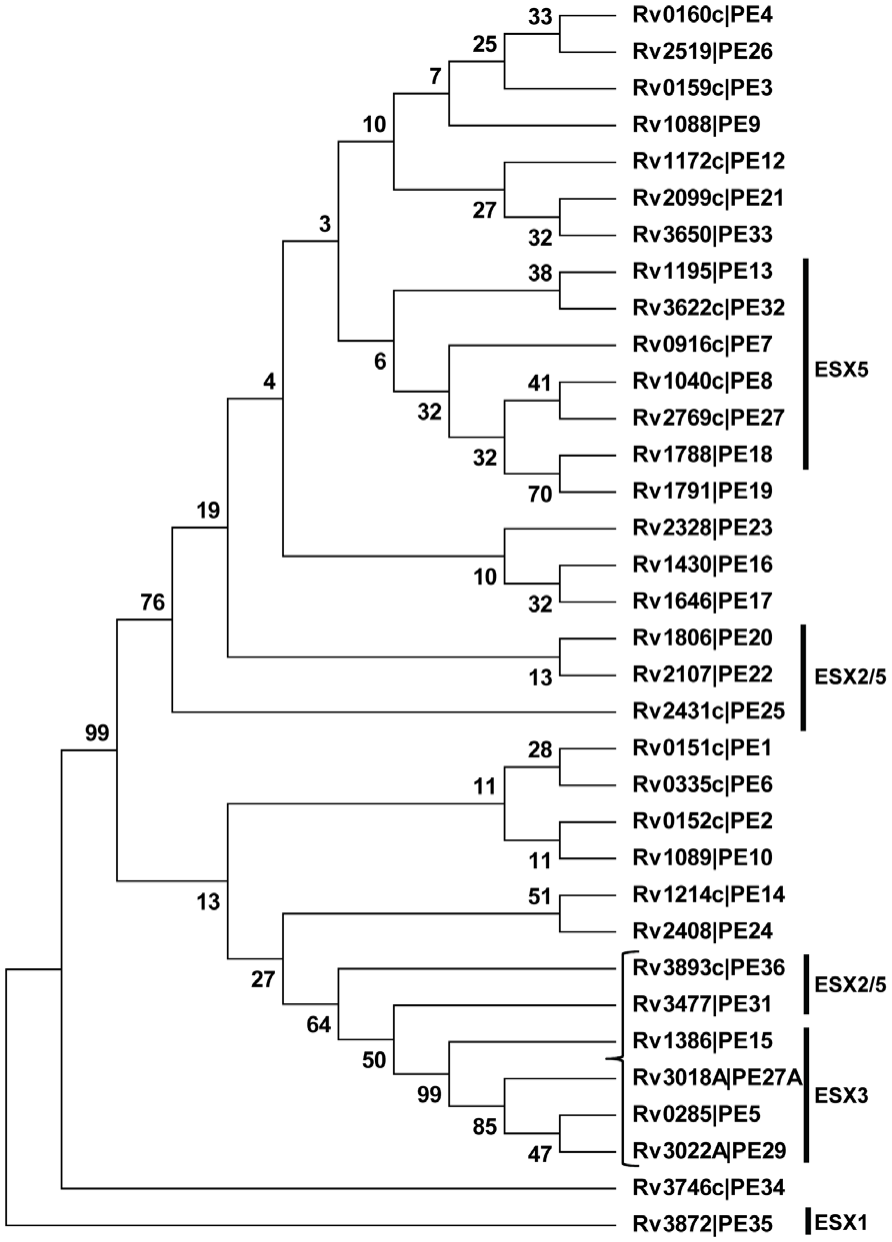
Phylogenetic analysis of the PE sub-family *of M.tb*. (A) Bootstrap consensus tree of the PE sub-family which includes 34 proteins of the ‘PE only’ and ‘PE with variable C-terminus’ classes rooted on PE35, highlighting clusters containing the ESX associated PE proteins. The percentage of replicate trees in which the associated taxa clustered together in the bootstrap test is shown next to the branches. The tree is drawn to scale, with branch lengths in the same units as those of the evolutionary distances used to infer it. The bracket shows the cluster chosen for analysis.

**Figure 2:**
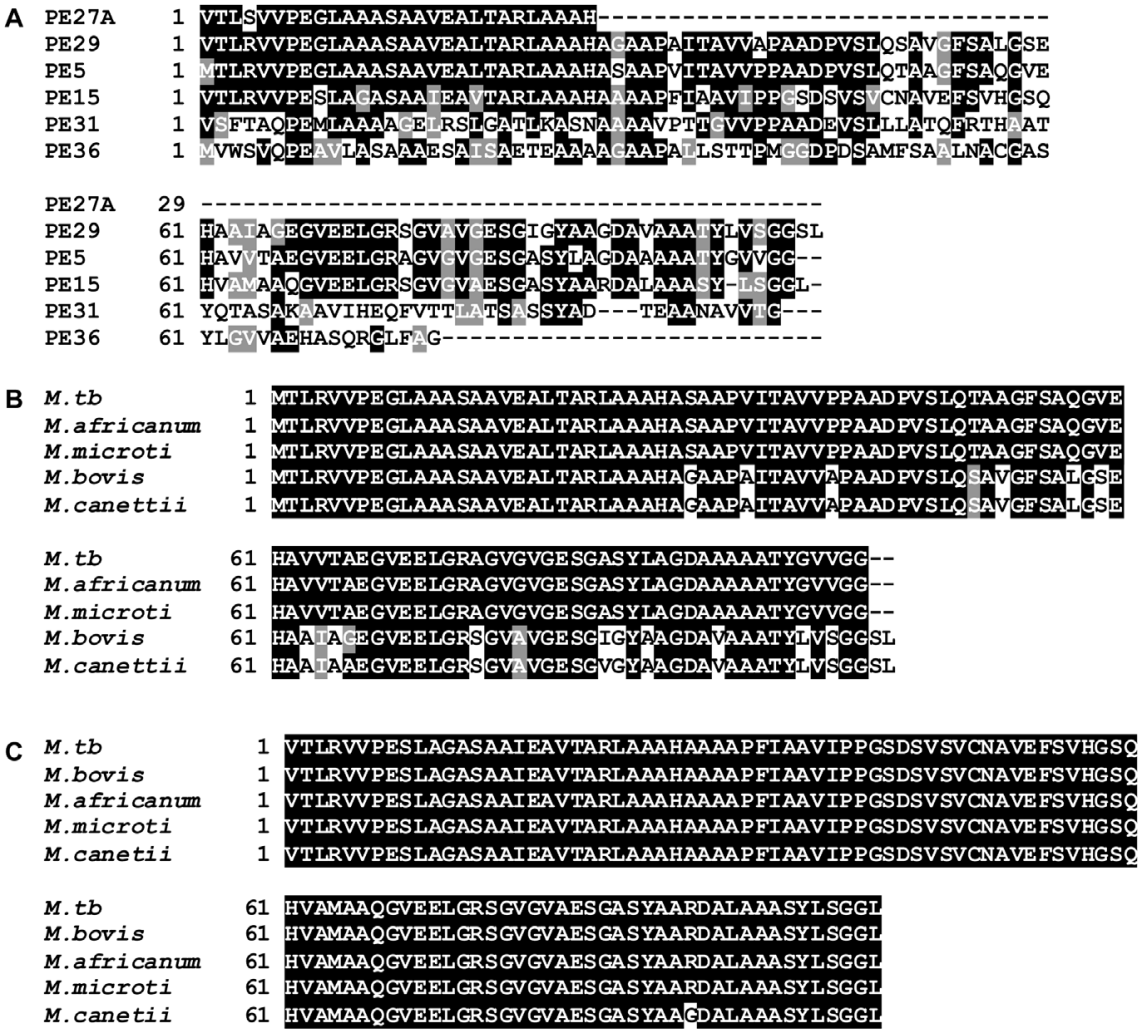
Sequence conservation of *M.tb* PE5 and PE15. (A) Multiple sequence alignment of the 6 proteins in the indicated cluster in Figure 1. Multiple sequence alignment of protein sequences of PE5 (B) and PE15 (C) from members of the *M.tb* complex.

As a prelude to its functional characterization, we examined the evolutionary relationships among the PE protein sub-family which includes 34 proteins of the ‘PE only’ and ‘PE with variable C-terminus’ classes, by phylogenetic analysis. As shown in the dendrogram (Figure 1) rooted on PE35, all the *esx* associated PE proteins mapped to three distinct clusters. For functional analyses, we chose the *esx*-3 associated proteins PE5 (Rv0285) and PE15 (Rv1386) present in the cluster closest to the root as representative members of this family. These two proteins have previously been assigned to a common sublineage in a phylogenetic reconstruction of the 110 aa N-terminal domains of all the PE proteins [3]. Multiple sequence alignment of the proteins in this cluster revealed a high degree of sequence similarity (Figure 2A), suggesting that these six proteins might be functionally related. A recent analysis performed to detect the frequency and nature of genetic variation in the *PE* and *PPE* genes using genome sequences of the *M.tb* complex, has revealed that the frequency of non-synonymous variations in the genes of this cluster are either limited or absent [27]. The strong selection against the accumulation of mutations in these genes implies an essential role for these proteins in mycobacterial physiology. PE5 and PE15 share >75% similarity and are both highly conserved throughout the *M.tb* complex (Figure 2B and C).

### *M.smegmatis* Strains Expressing *PE5* and *PE15* Show Enhanced Survival in Macrophages

**Figure 3:**
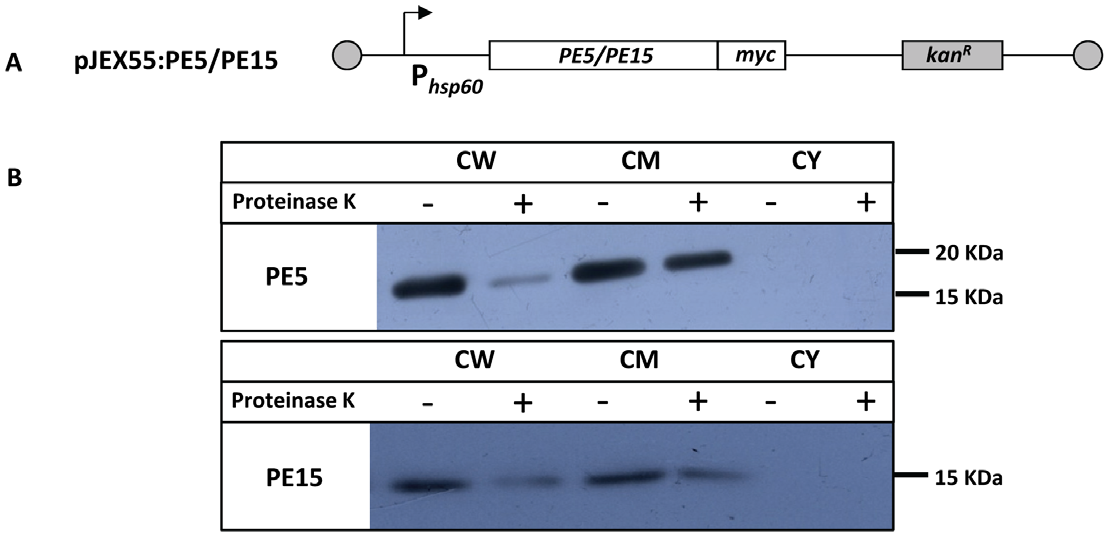
Sub-cellular localization and surface accessibility. (A) Schematic representation of the c-myc fusion constructs of PE5/PE15 generated in the episomal plasmid pJEX55 under the control of the constitutive *hsp60* promoter. (B) Immuno-detection of PE5 and PE15 in sub-cellular fractions of Proteinase K treated recombinant *M.smegmatis* strains expressing PE5-myc and PE15-myc. All proteins were detected using an anti c-myc mAb. CW - cell wall fraction, CM - cell membrane fraction, CY - cytoplasmic fraction.

**Figure 4:**
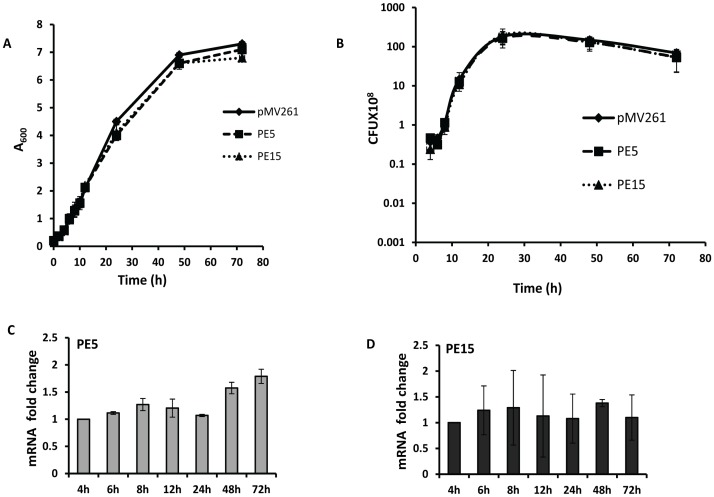
Gene expression analysis and growth of recombinant *M.smegmatis* expressing *PE5* and *PE15*. *In vitro growth* profiles of *M.smegmatis* expressing pMV261, PE5 and PE15 generated by measuring OD (A) and enumeration of CFU counts (B). Real time RT-PCR quantitation of PE5 (C) and PE15 (D) transcripts as a function of growth. Transcript levels of are represented relative to mRNA levels of the cognate gene at 4 h which is assigned a value of 1. Error bars represent ± SEM.

**Figure 5:**
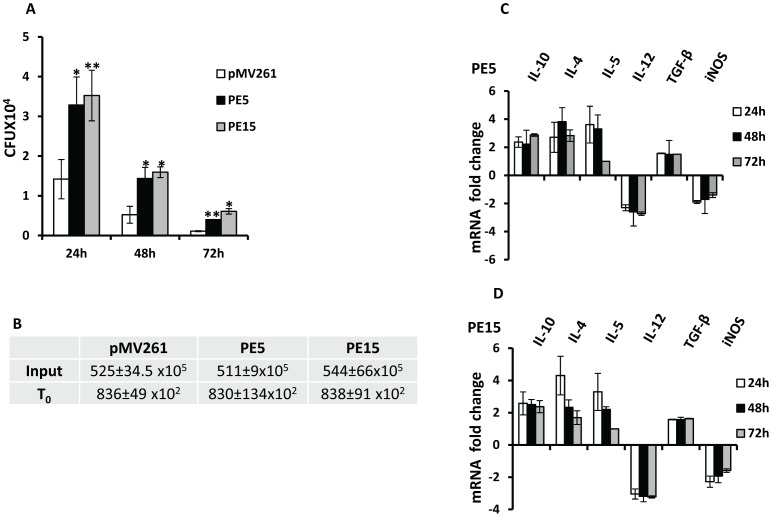
Physiology of recombinant *M.smegmatis* strains expressing *PE5* and *PE15* in resting J774.1 macrophages. (A) CFU counts of *M.smegmatis* expressing empty vector (pMV261), *PE5* (PE5) and *PE15* (PE15) 24, 48 and 72 h post infection. (B) Input and T_0_ (post-infection) CFU counts of infecting bacilli (± SEM). (C and D) Transcript levels of pro- and anti-inflammatory cytokines and *iNOS*, 24, 48 and 72 h post infection, p<0.05 for all data points. Error bars represent ± SEM from three biological replicates. **p<0.005, *p<0.05.

**Figure 6:**
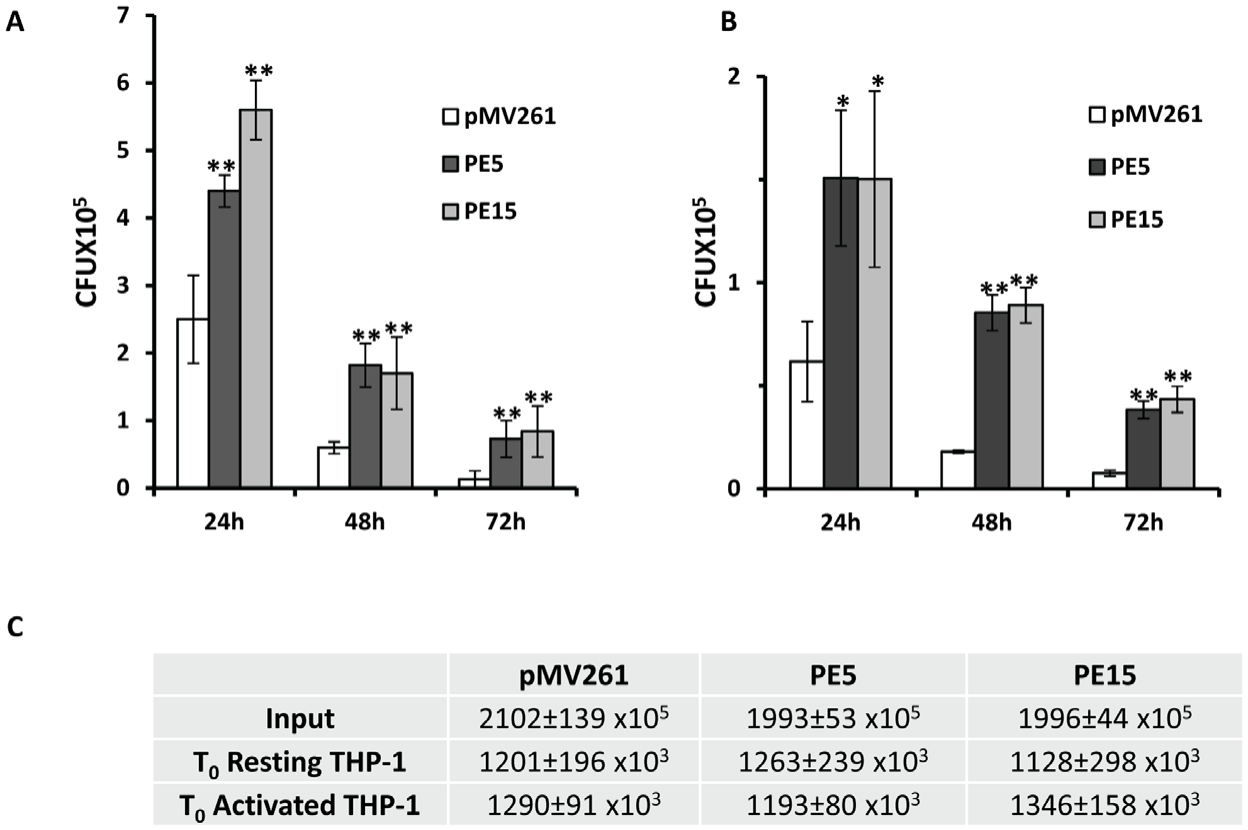
Viability of *M.smegmatis* strains expressing *PE5* & *PE15* in THP-1 macrophages. CFU counts of *M.smegmatis* expressing empty vector (pMV261), *PE5* (PE5) and *PE15* (PE15) in resting (A) and activated THP-1 macrophages (B) 24, 48 and 72 h post infection. (C) Input and T_0_ (post-infection) CFU counts of infecting bacilli (± SEM). Error bars represent ± SEM from three biological replicates. **p<0.005, * p<0.05.

To investigate the role of PE5 and PE15 in mycobacterial physiology, we used the saprophyte *M.smegmatis*, which has been extensively employed as a surrogate model to study the functions of this family of proteins [9], [28], [29], [30], [31], [32], [33], [34], [35]. Sub-cellular fractionation of recombinant *M.smegmatis* expressing c-myc fusions of PE5 and PE15 (schematic representation shown in Figure 3A) showed that these proteins were associated with the cell envelope (Figure 3B). Equal loading was ensured for each fraction (Figure S1) and appropriate controls were included to ensure the purity of the sub-cellular fractions (Figure S2) as described [9]. The exposure of these proteins on the cell surface was assessed by Proteinase K treatment with (Figure 3B) or without (Figure S3A) sub-cellular fractionation. The decrease in the western blot signal corresponding to the two proteins following this treatment indicated that they were indeed cell-surface exposed. No decrease was observed in the signal corresponding to the cytoplasmic control ΔNCMPT64 (MPT64 lacking the N-terminal signal sequence [9]) following Proteinase K treatment (Figure S3B), validating the specificity of this technique. This observation was consistent with *in silico* prediction of trans-membrane domains (Figure S4A) and hydrophobicity of these proteins (Figure S4B). Cell surface associated proteins are important modulators of pathogenic processes and are believed to mediate their biological functions by interacting with host cells. To test the possible role of PE5 and PE15 in mediating host-pathogen interactions, these genes were expressed from the constitutive *hsp60* promoter in *M.smegmatis* using the shuttle vector pMV261 [26]. Growth profiles of *M.smegmatis* strains expressing *PE5*, *PE15* and the empty vector were comparable, both by OD measurement (Figure 4A) as well as CFU (Figure 4B) enumeration, suggesting that over-expression of these genes does not lead to growth defects *in vitro*. Real time RT-PCR analysis of recombinant *M.smegmatis* strains using gene specific primers (Table S1) was used to confirm the expression of both genes, and the transcript profiles suggested that the expression of these genes did not vary as a function of growth (Figure 4C and D). Although it lacks homologues of most of the PE-PPE family proteins, a possible counterpart of PE5/PE15 is present in the *M.smegmatis* genome (MSMEG_0618). No amplification products were observed in the negative controls of this experiment, suggesting that the transcript levels estimated were specific to *M.tb* PE5/PE15 and were not influenced by any mRNA species produced from this homologue (data not shown). To ensure that the activity of MSMEG_0618 did not influence the outcome of our study an *M.smegmatis* strain expressing the empty vector pMV261 was used as a baseline control for all subsequent experiments. To determine the possible role of the test proteins in intracellular persistence, we examined the *in vivo* survival of the above recombinant *M.smegmatis* strains in a macrophage model of infection. Strains expressing *PE5* and *PE15* showed significantly higher bacillary counts in both resting J774.1 (Figure 5A) and THP-1 (Figure 6A) macrophages 24, 48 and 72 hours post infection, in comparison to the empty vector control. The same phenotype was observed in activated THP-1 macrophages as well, although overall CFU counts were lower in comparison to resting THP-1 cells (Figure 6B). The equal input as well as T_0_ counts of infecting bacilli, indicated that the observed increase in intracellular CFU counts was not a consequence of differential bacillary uptake by macrophages (Figure 5B and 6C).

### PE5 and PE15 Alter the Innate Immune Response in Macrophages

**Figure 7:**
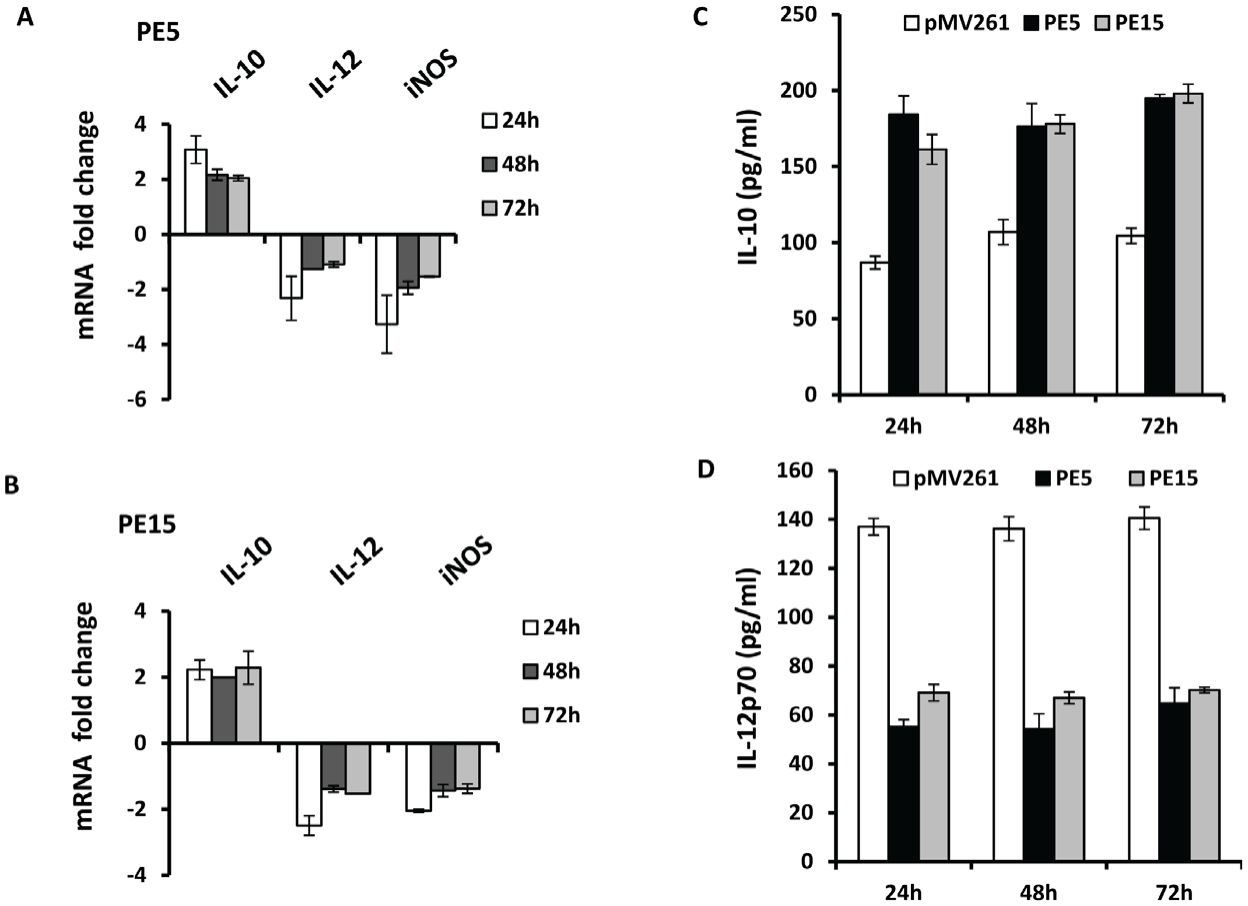
*In vivo* effects of THP-1 macrophage infection with *M.smegmatis* strains expressing *PE5* and *PE15*. (A) and (B) Transcript levels of IL-10, IL-12 and *iNOS,* in resting macrophages infected with *M.smegmatis* expressing empty vector (pMV261), *PE5* (PE5) and *PE15* (PE15) 24, 48 and 72 h post infection, p<0.05 for all data points. IL-10 (C) and IL-12 (D) levels in the culture supernatants of infected cells 24, 48 and 72 h post infection, p<0.005 for all data points. Error bars represent ± SEM from three biological replicates.

To identify factors contributing to the increased intra-macrophage survival of the recombinant *M.smegmatis* strains, we measured the levels of inducible nitric oxide synthase 2 (*iNOS*), which is a key determinant of intracellular bacillary burden in host cells [36], [37], [38]. Following infection, we observed a down-regulation of the *iNOS* transcript in both J774.1 (Figure 5C and D) as well as in infected THP-1 macrophages (Figure 7A and B), 24, 48 and 72 h post infection. To assess the immuno-modulatory potential of PE5 and PE15, we estimated the levels of several cytokines known to regulate the intracellular fate of *M.tb* [39]. We observed a reduction in transcript levels of IL-12, a key pro-inflammatory cytokine and a significant up-regulation in the levels of the transcripts of the anti-inflammatory cytokines IL-10, IL-4, IL-5 and TGF-β in infected J774.1 cells (Figure 5C and D) at all the above time points. Infected THP-1 macrophages showed up- and down- regulation of transcripts corresponding to IL-10 and IL-12 respectively (Figure 7A and B). In addition, the secreted levels of IL-10 and IL-12 in the culture supernatants of these cells were found to mirror the changes observed at the transcript level at the indicated time points (Figure 7C and D).

### PE5 and PE15 Trigger the Activation of MAP Kinases Required for IL-10 Production

**Figure 8:**
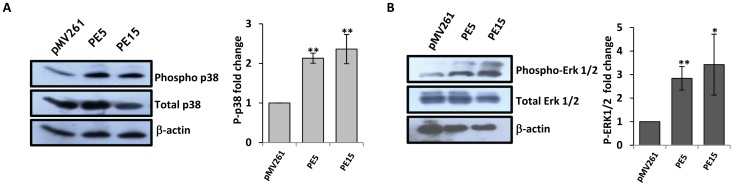
MAP kinase signalling and its correlation to IL-10 levels in infected THP-1 macrophages. Phosphorylation levels of p38 (A) and ERK1/2 (B) and their densitometric quantitation from resting THP-1 cells infected with *M.smegmatis* expressing pMV261, *PE5* and *PE15* 24 h post infection. Error bars represent ± SEM of three biological replicates. **p<0.005, *p<0.05. The western blots shown are representative of at least two biological replicates.

**Figure 9:**
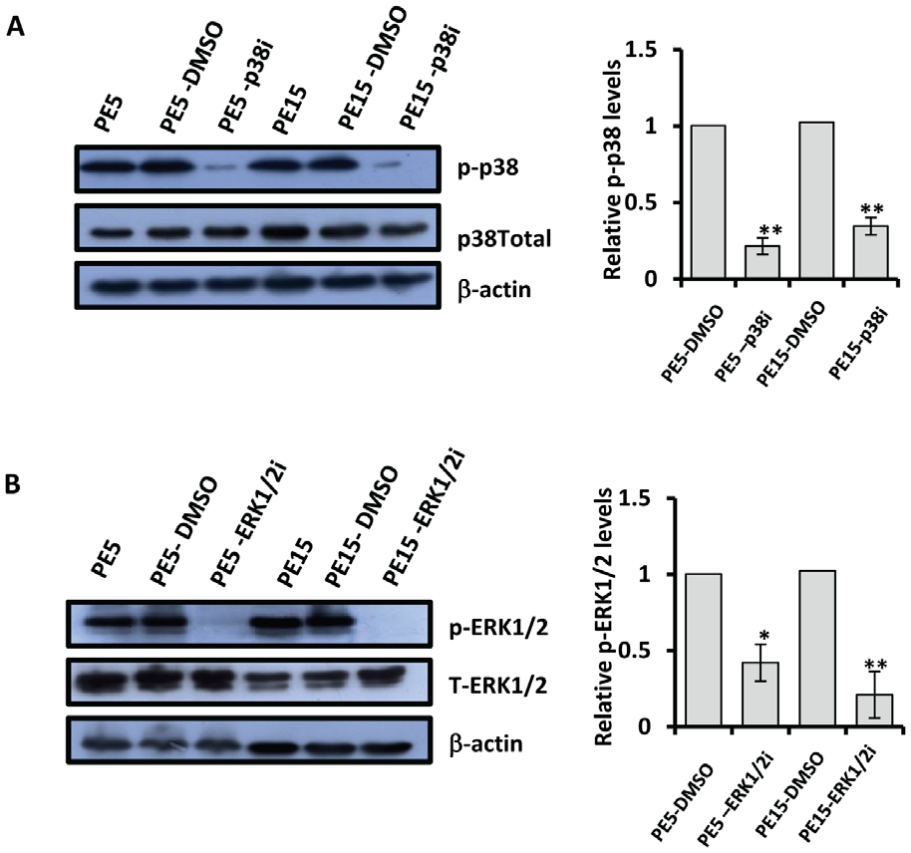
Inhibition of MAP kinase signalling in infected THP-1 macrophages. Phosphorylation levels of p38 (C) and ERK1/2 (D) and their densitometric quantitation from resting THP-1 cells infected with *M.smegmatis* expressing pMV261, PE5 and PE15 24 h post infection in the presence of p38 and ERK1/2 inhibitors. Error bars represent ± SEM of three biological replicates. **p<0.005, *p<0.05. The western blots shown are representative of at least two biological replicates.

**Figure 10:**
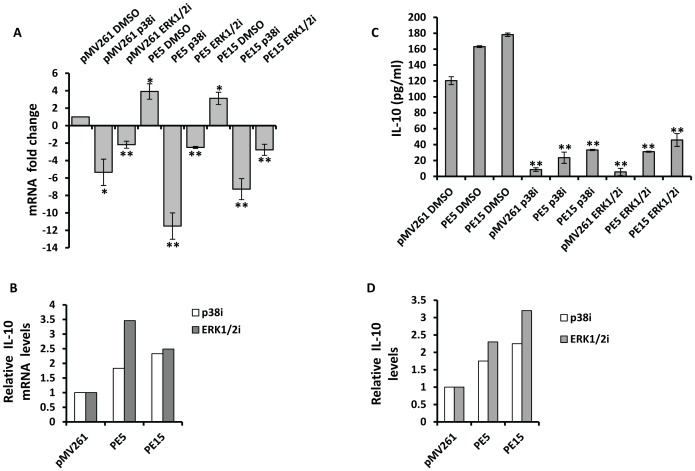
Effect of MAP kinase inhibition on IL-10 expression. (A) Real time RT-PCR quantitation of IL-10 transcripts in resting THP-1 cells infected with *M.smegmatis* expressing pMV261, PE5 and PE15 24 h post infection in the presence of p38 and ERK1/2 inhibitors. Transcript levels of IL-10 in infected DMSO treated cells are depicted relative to the empty vector control which is assigned a value of 1. Fold changes in mRNA levels of each inhibited sample are represented relative to the cognate DMSO treated control. (C) IL-10 protein levels from the culture supernatant of THP-1 infected with *M.smegmatis* expressing empty vector, PE5 and PE15 24 h post infection. (B) and (D) are histograms derived from the data in (A) and (C) respectively that represent IL-10 levels following inhibition, relative to the empty vector controls. Error bars represent ± SEM of at least two biological replicates. **p<0.005, * p<0.05.

Activation of the MAP kinase pathway is critical for the induction of IL-10 in macrophages [40]. We investigated the likely role of PE5 and PE15 in stimulating the phosphorylation of p38 and ERK1/2, the primary effectors in this signaling cascade. Infection of resting THP-1 macrophages with *PE5* and *PE15* expressing *M.smegmatis* led to the induction of the p38 and ERK1/2 pathways as indicated by increased phosphorylation of p38 and ERK1/2 (Figure 8A and B) 24 h post infection. This observation was corroborated by using the pharmacological inhibitors SB203580 and PD98059 which specifically target the p38 and ERK1/2 pathways respectively. As shown in Figure 9A and B, treatment with these compounds reduced the phosphorylation levels of p38 and ERK 1/2 and also resulted in the inhibition of IL-10 production both at the transcript and protein levels (Figure 10A and C). The reduced fold decrease in IL-10 levels relative to the *M.smegmatis* vector control infected cells was indicative of the specificity of the PE5/PE15 mediated expression of IL-10 via the MAP Kinase pathway (Figure 10B and D).

### *PE5-PPE4* and *PE15-PPE20* are Co-operonic Gene Pairs but their Gene Products do not Physically Interact

**Figure 11:**
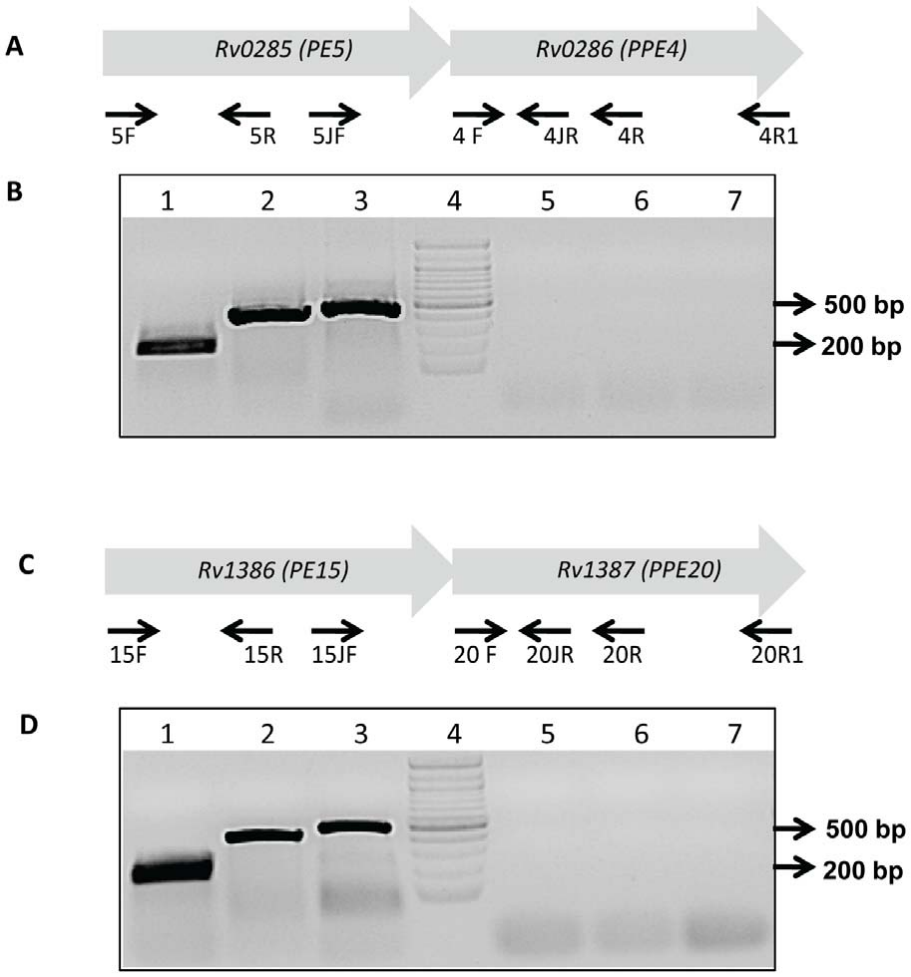
RT-PCR analysis of the putative co-operonic pairs *PE5-PPE4* and *PE15-PPE20*. Schematic representation of the genomic organization of the *PE5* (A) and *PE15* (C) loci in the *M.tb* genome showing the positions of the primers used in the study. (B) RT-PCR amplification products of the *PE5-PPE4* gene pair: Lane 1- (5F+5R), Lane 2- (5JF+4JR), Lane 3 - (4F+4R), Lanes 5, 6 and 7 correspond to -RT controls for these respective primer pairs, Lane 4–100 bp DNA ladder (D) RT-PCR amplification products of the *PE15-PPE20* gene pair: Lane 1- (15F+15R), Lane 2- (15JF+20JR), Lane 3 - (20F+20R); Lanes 5, 6 and 7 correspond to -RT controls for these respective primer pairs. Lane 4–100 bp DNA ladder.

The *M.tb PE5* and *PPE4* genes are located adjacent to each other in the same orientation with an intergenic distance of 2 bp. Similarly, *PE15* and *PPE20* share a 4 bp overlap. Both these gene pairs are among the 14 *PE-PPE* gene pairs predicted to be co-operonic [5]. To verify this hypothesis we performed RT-PCR analyses in *M.tb* H37Ra using primer pairs (Table S1) designed to amplify ORF and junction specific regions of the two gene pairs (Figure 11A and C). Based on the sizes of amplicons obtained as expected, and the absence of amplification products in the -RT control lanes we concluded that these pairs were indeed transcribed as mono-cistronic messages (Figure 11B and D), validating the above prediction. Since the *PE25-PPE41* pair has earlier been reported to be co-operonic and also to physically interact to form a stable complex [4], [41], [42], we tested the possible interaction of the PE5-PPE4 and PE15-PPE20 protein pairs using the mycobacterial protein fragment complementation (M-PFC) assay [43]. Co-transformants of *M.smegmatis* mc^2^155 expressing the two protein pairs fused independently to the murine dihydrofolate reductase domains F [1], [2] and F [3], did not show any resistance to trimethoprim (data not shown), suggesting that these cognate protein pairs do not physically interact *in vivo*.

## Discussion

While the functions of some proteins of the PPE and PE_PGRS sub-families have been deciphered for their roles in host immune regulation [44], the involvement of the ‘PE only’ sub-group in this process has remained largely undefined. To fill this lacuna we examined the physiological roles of PE5 and PE15, two representative members of this sub-family associated with the *esx*-3 gene cluster. Both these proteins are surface localised or exported in *M.tb* [45], and their transcripts are induced under hypoxia [19]. We observed that recombinant *M.smegmatis* strains expressing *PE5* and *PE15* showed enhanced survival in both murine and human macrophage cell lines indicating a potential role for these proteins in bacterial persistence. Since we observed these proteins to be localised to the cell envelope in *M.smegmatis*, it was conceivable that they could be involved in mediating events at the mycobacterium-host interface leading to enhancement in bacillary survival. Macrophages infected with these recombinant strains were associated with decreased transcript levels of *iNOS*, lower levels of pro- and increased levels of anti-inflammatory cytokines. All these are consistent with the strategies used by *M.tb* to subvert macrophage control of intracellular bacillary loads [36], [37], [38], [39]. The balance between pro- and anti-inflammatory components of the human immune system is dynamic and constantly subject to change. Th1-type cells secrete high levels of the pro-inflammatory cytokines IL-12, TNF-α and interferon-gamma which activate macrophages and promote cell-mediated immune responses against intra-cellular pathogens like *M.tb* [46], [47]. IL-4, IL-5, IL-10 and transforming growth factor-β are anti-inflammatory cytokines, part of the Th2 type response which favours survival of the pathogen. IL-10 inhibits the production of host-protective pro-inflammatory cytokines [48] and is an inhibitor of early mycobacterial clearance [49]. We consistently observed a decrease in levels of IL-12 along with increased IL-10 transcript levels in infected macrophages. Similar to this finding, infection of THP-1 macrophages with a recombinant *M.smegmatis* strain expressing PPE18 led to TLR2 dependent induction of IL-10 [32]. Analysis of flux through the MAP kinase pathway in infected macrophages identified a role for p38 and ERK 1/2 in IL-10 induction by recombinant *M.smegmatis* strains expressing *PE5* and *PE15*. Pharmacological inhibition of either ERK or p38 activation led to a reduction, but not abrogation of IL-10 expression, suggesting that these two pathways might play cooperative roles in this context. Our observation that *PE5* and *PE15* are co-transcribed with their respective downstream genes *PPE4* and *PPE20* raises the possibility that the two gene pairs may perform co-ordinate functions. However, the respective protein pairs were observed not to interact in the *in vivo* M-PFC assay, implying a lack of direct physical association between them. It has been suggested that *PE-PPE* gene pairs may be unstable when expressed singly [42]. On the contrary, we were able to purify both PE5 and PE15 from the soluble fractions of *E.coli* strains over-expressing these proteins (Figure S5), suggesting that this may not be true of all PE-PPE protein pairs.

**Figure 12:**
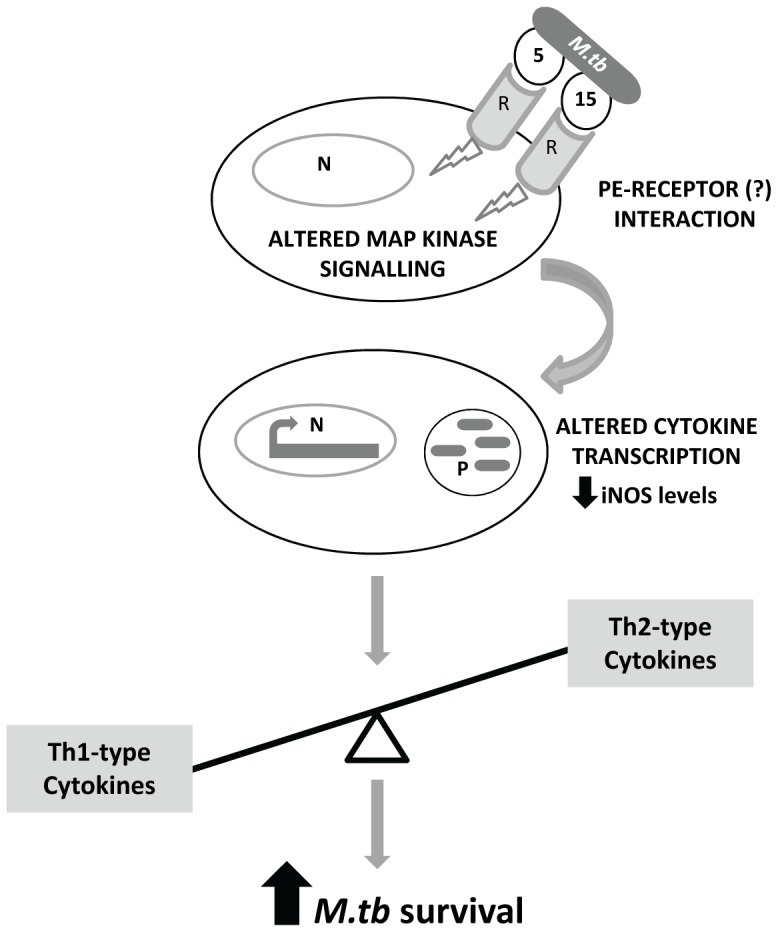
Model depicting the possible role of PE5 (5) and PE15 (15) in immune subversion by *M.tb*. N-Nucleus, P-Phagosome, R-Receptor.

These observations strongly suggest that PE5 and PE15 are immuno-modulatory proteins and are likely to participate in either establishment or maintenance of infection by shifting the Th1/Th2 balance to favour *M.tb* infection via altered MAP Kinase signalling (Figure 12). To the best of our knowledge, this is the first report of such a property for the ‘PE only’ sub-group of the PE/PPE family of proteins. Since their N-termini show extensive homology (Figure S6) this finding may be indicative of similar functions for the 32 other proteins in this sub-group as well. Our observations also raise several questions regarding the functioning of these proteins. What receptor(s) do these proteins bind on the host cell surface to induce their downstream effects? The PE/PPE proteins PE_PGRS11, PE_PGRS33 and PPE18 have been demonstrated to function in a TLR2 dependent manner [16], [28], [32], but it remains to be established if this is a general property of this family. Since multiple proteins of the PE/PPE family perform immuno-modulatory roles through conserved signaling pathways, this could (mis)lead us to conclude that functional redundancy is a norm within this family. However, the expression of several PE/PPE genes has been observed to vary in response to conditions relevant to *M.tb* physiology including hypoxia, non-replicative persistence, diamide treatment, oxidative stress, iron depravation and starvation [19]. This suggests that contrary to being redundant these proteins may play stage specific roles in modulating disease pathogenesis depending on the microenvironmental niche of the pathogen. Experiments to identify the receptor(s) to which PE5 and PE15 bind, are currently in progress. We are also considering the possibility that these and other PE proteins may function synergistically in the pathogenic process. The implications of the association of *PE5* and *PE15* (and other *PE-PPE* gene pairs) within *esx* clusters remain unclear. It has been speculated that PE/PPE complexes or the individual constituent proteins could be virulence effectors secreted by either their cognate or non-cognate ESX systems [50]. A recent study identified the YxxxD/E motif as a general secretion signal present in all known mycobacterial TypeVII secretion system substrates or substrate complexes [51]. Since this signature is also conserved in both PE5 and PE15, it is pertinent to examine this hypothesis by monitoring the secretion of these proteins in *M.tb* strains carrying mutations in the *esx* genes.

In summary, our findings greatly expand the repertoire of proteins that *M.tb* utilises to alter host immune responses. Functional dissection of the ‘PE only’ sub-group of proteins is likely to elucidate the complexities of the immuno-modulatory mechanisms utilised by *M.tb*, and could help in the design of future therapeutic strategies for TB control.

## Supporting Information

Figure S1 **Coomassie stained SDS PAGE profiles showing equal loading for the sub-cellular fractions of Proteinase K treated** ***M.smegmatis*** **expressing PE5 and PE15.**
(TIF)

Figure S2 **Immunoblot analysis to assess purity of sub-cellular fractions of recombinant** ***M.smegmatis*** **strains expressing PE11-myc (cell wall control), N-terminal HtrA-myc (cell membrane control) and ΔNCMPT64-myc (cytosolic control).** All proteins were detected using an anti c-myc mAb. Coomassie stained SDS PAGE profiles showing equal loading for the respective subcellular fractions are shown below the blots. CW - cell wall fraction, CM - cell membrane fraction, CY - cytoplasmic fraction.
(TIF)

Figure S3 **Proteinase K treatment of** ***M.smegmatis*** **expressing PE5, PE15 and ΔNCMPT64.** Western blot analysis of Proteinase K treated *M.smegmatis* expressing PE5-myc, PE15-myc (A) and ΔNCMPT64-myc (B), with their respective untreated controls. Coomassie stained gels are shown as representative of each Western blot for equal loading.
(TIF)

Figure S4 ***In silico*** **protein sequence analysis of** ***M.tb*** **PE5 and PE15.** Transmembrane prediction (A), and Hydrophobicity (B) analyses of PE5 and PE15.
(TIF)

Figure S5 **Expression of recombinant** ***M.tb*** **PE5 and PE15 in** ***E.coli*** **.** SDS-PAGE purification profiles of 6XHIS-tagged PE5 (A) and PE15 (B). Lane1: Induced cell lysate, Lane2: Protein size marker, Lane 3: purified proteins (arrowheads)
(TIF)

Figure S6 **Sequence conservation among the PE subfamily of** ***M.tb*** **.** Multiple sequence alignment of N-termini of 31/34 proteins of the ‘PE only’ sub-family; PE1, PE16 and PE24 were omitted from the alignment for illustrative purposes.
(TIF)

Table S1 **Oligonucleotides used in this study.**
(DOCX)
