# Supplementary figures and images for: The Mycobacterium tuberculosis PE Proteins Rv0285 and Rv1386 Modulate Innate Immunity and Mediate Bacillary Survival in Macrophages
Source: PLoS One. 2012 Dec 17;7(12):e51686. doi: 10.1371/journal.pone.0051686 (PMC3524191; doi:10.1371/journal.pone.0051686)

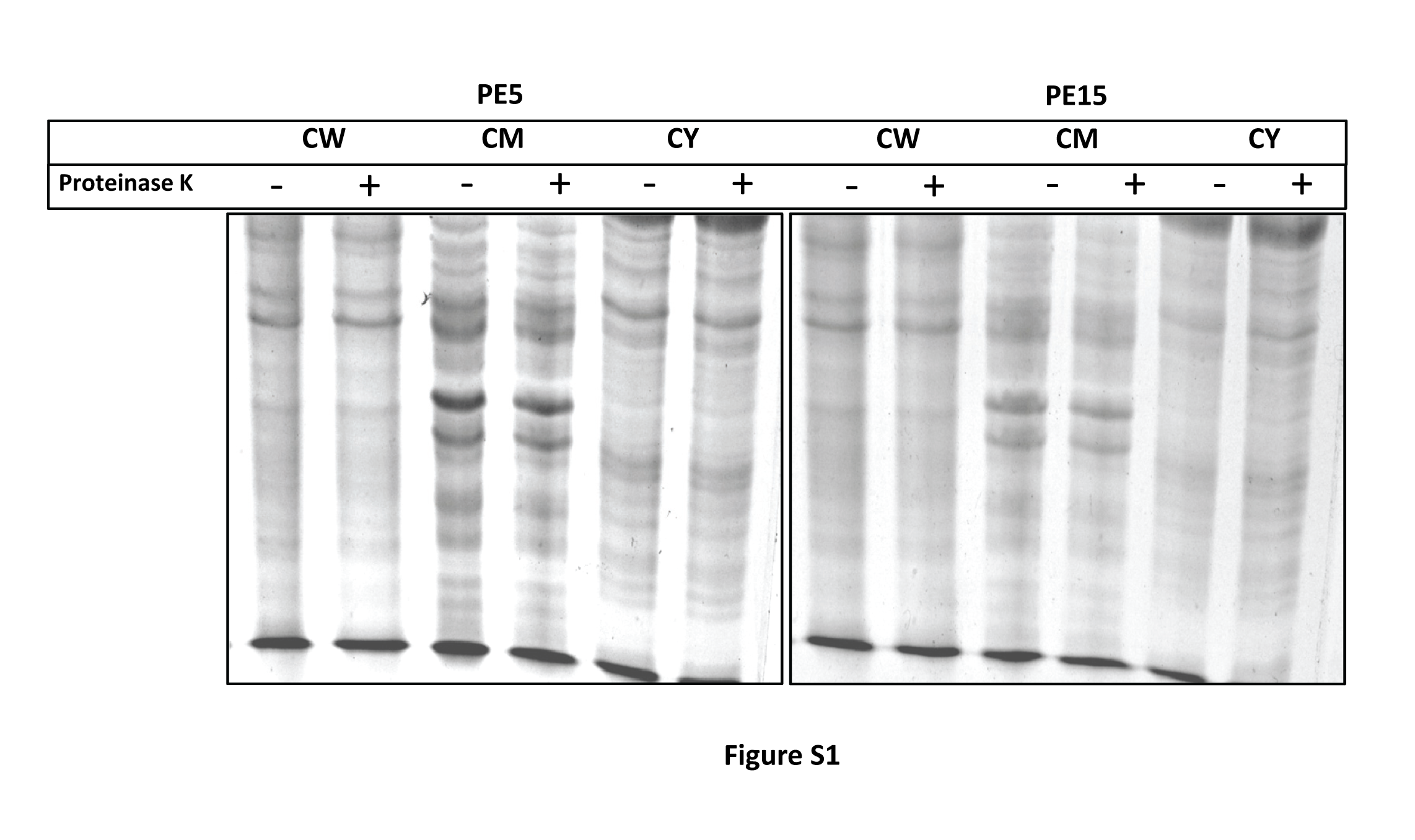

Supplement: Figure S1 — Coomassie stained SDS PAGE profiles showing equal loading for the sub-cellular fractions of Proteinase K treated M.smegmatis expressing PE5 and PE15. (TIF) [file pone.0051686.s001.tif]

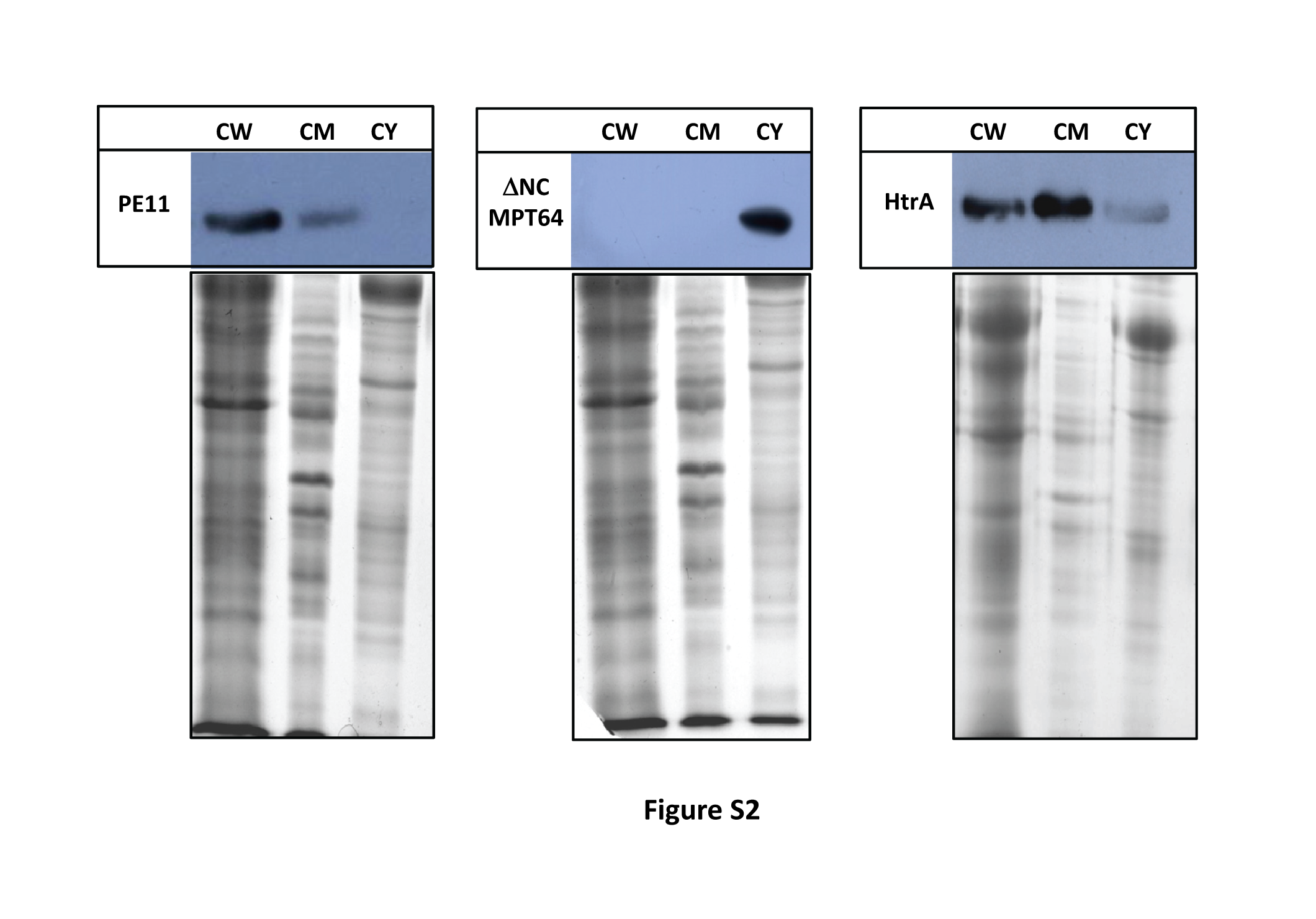

Supplement: Figure S2 — Immunoblot analysis to assess purity of sub-cellular fractions of recombinant M.smegmatis strains expressing PE11-myc (cell wall control), N-terminal HtrA-myc (cell membrane control) and ΔNCMPT64-myc (cytosolic control). All proteins were detected using an anti c-myc mAb. Coomassie stained SDS PAGE profiles showing equal loading for the respective subcellular fractions are shown below the blots. CW - cell wall fraction, CM - cell membrane fraction, CY - cytoplasmic fraction. (TIF) [file pone.0051686.s002.tif]

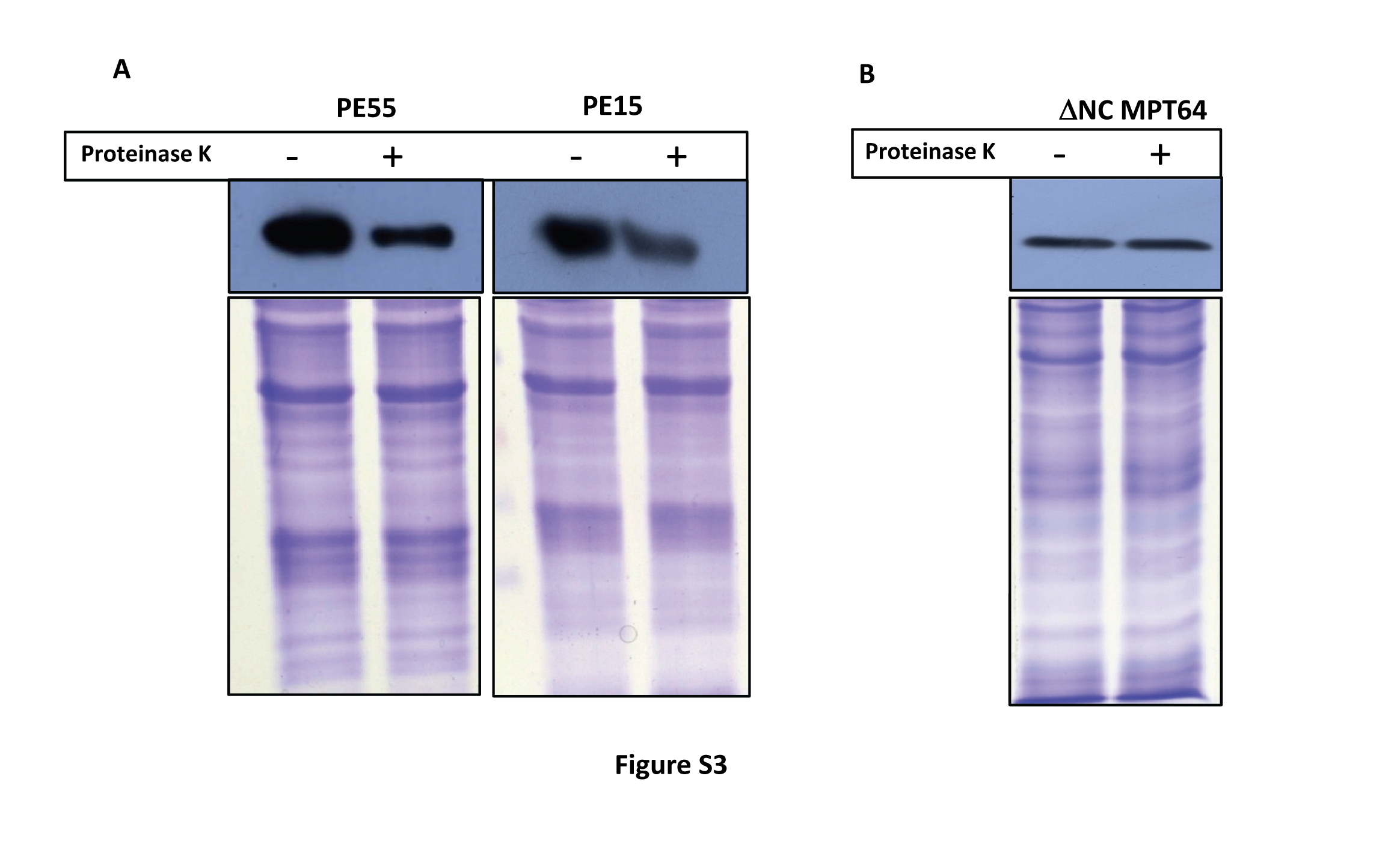

Supplement: Figure S3 — Proteinase K treatment of M.smegmatis expressing PE5, PE15 and ΔNCMPT64. Western blot analysis of Proteinase K treated M.smegmatis expressing PE5-myc, PE15-myc (A) and ΔNCMPT64-myc (B), with their respective untreated controls. Coomassie stained gels are shown as representative of each Western blot for equal loading. (TIF) [file pone.0051686.s003.tif]

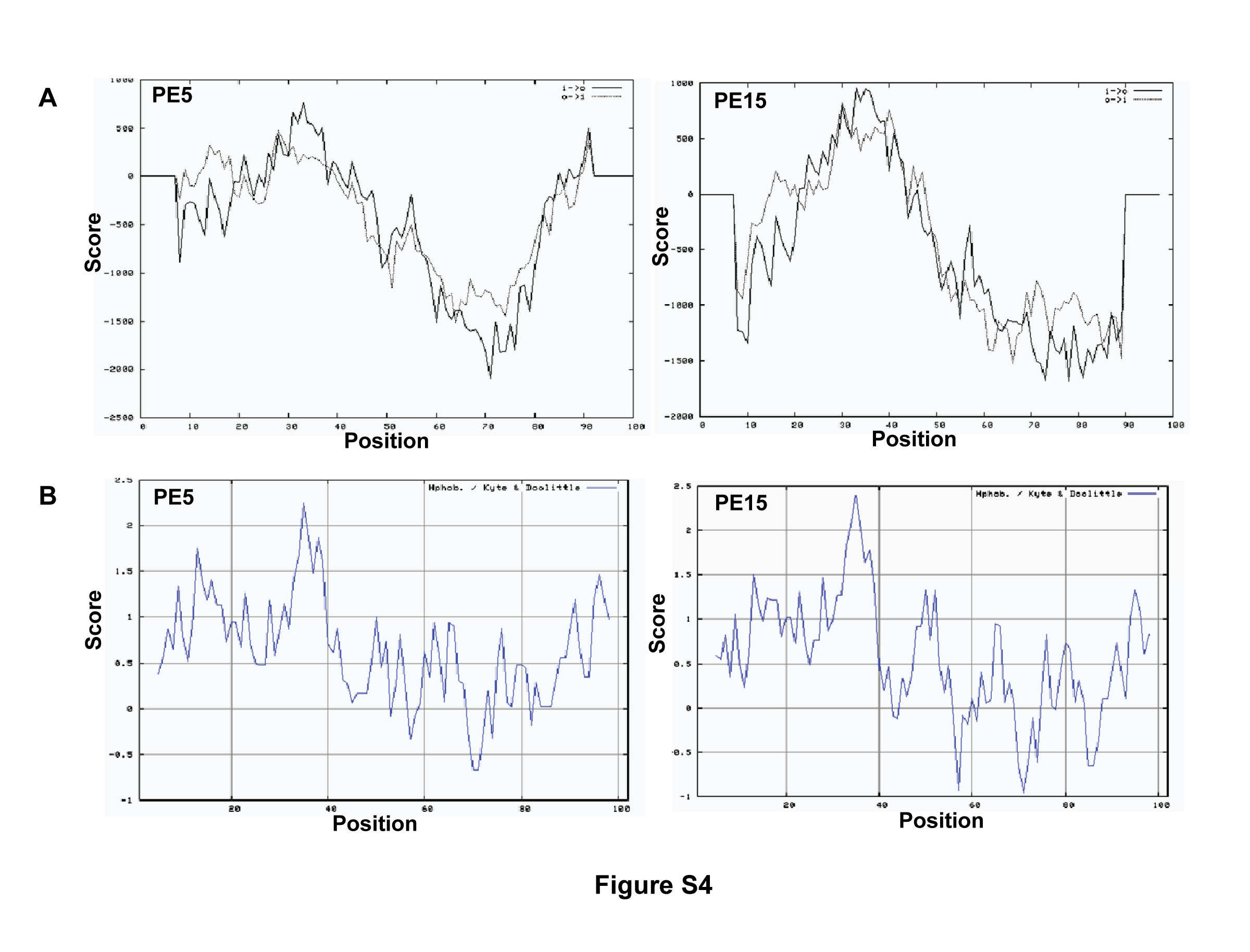

Supplement: Figure S4 — In silico protein sequence analysis of M.tb PE5 and PE15. Transmembrane prediction (A), and Hydrophobicity (B) analyses of PE5 and PE15. (TIF) [file pone.0051686.s004.tif]

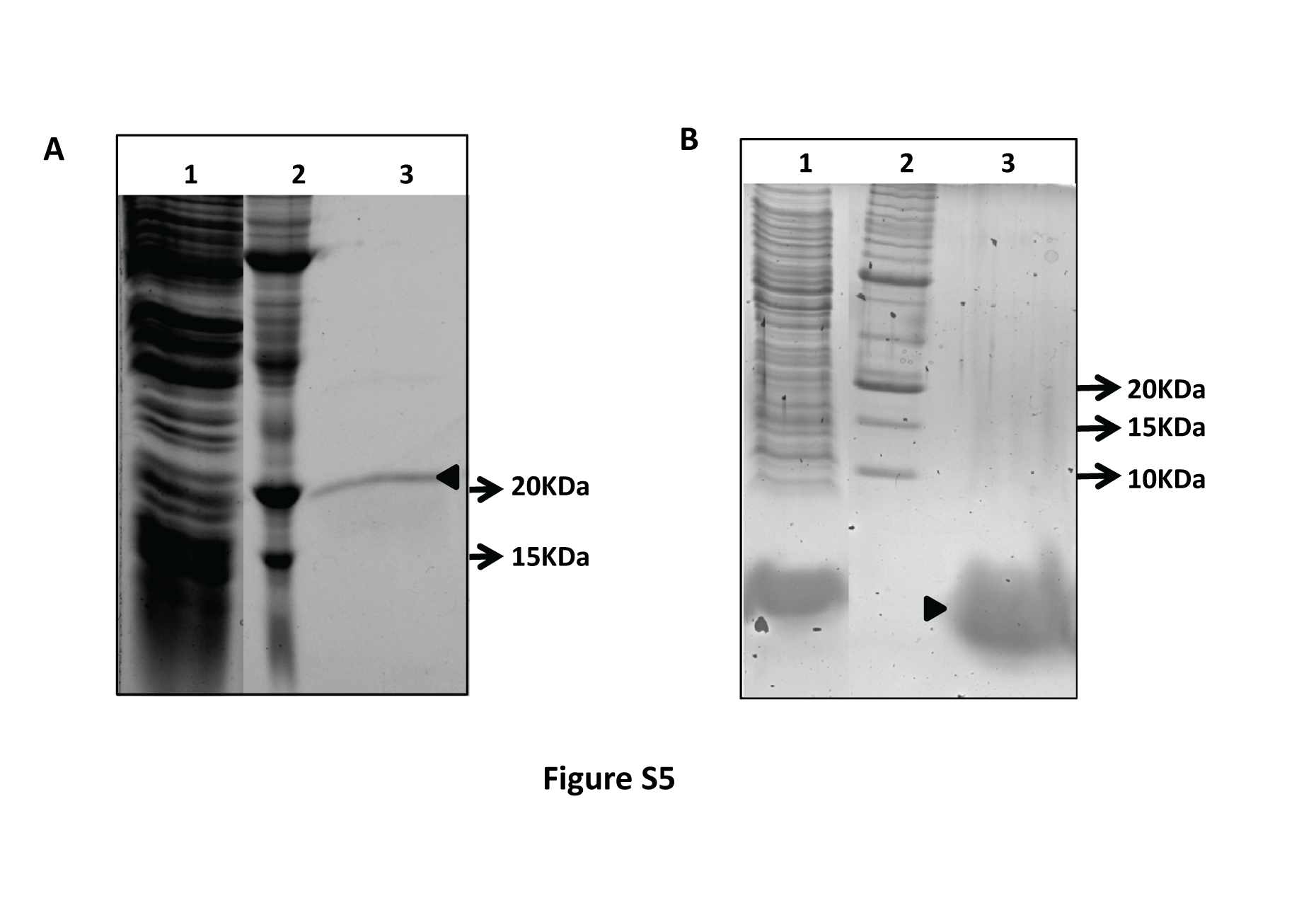

Supplement: Figure S5 — Expression of recombinant M.tb PE5 and PE15 in E.coli . SDS-PAGE purification profiles of 6XHIS-tagged PE5 (A) and PE15 (B). Lane1: Induced cell lysate, Lane2: Protein size marker, Lane 3: purified proteins (arrowheads) (TIF) [file pone.0051686.s005.tif]

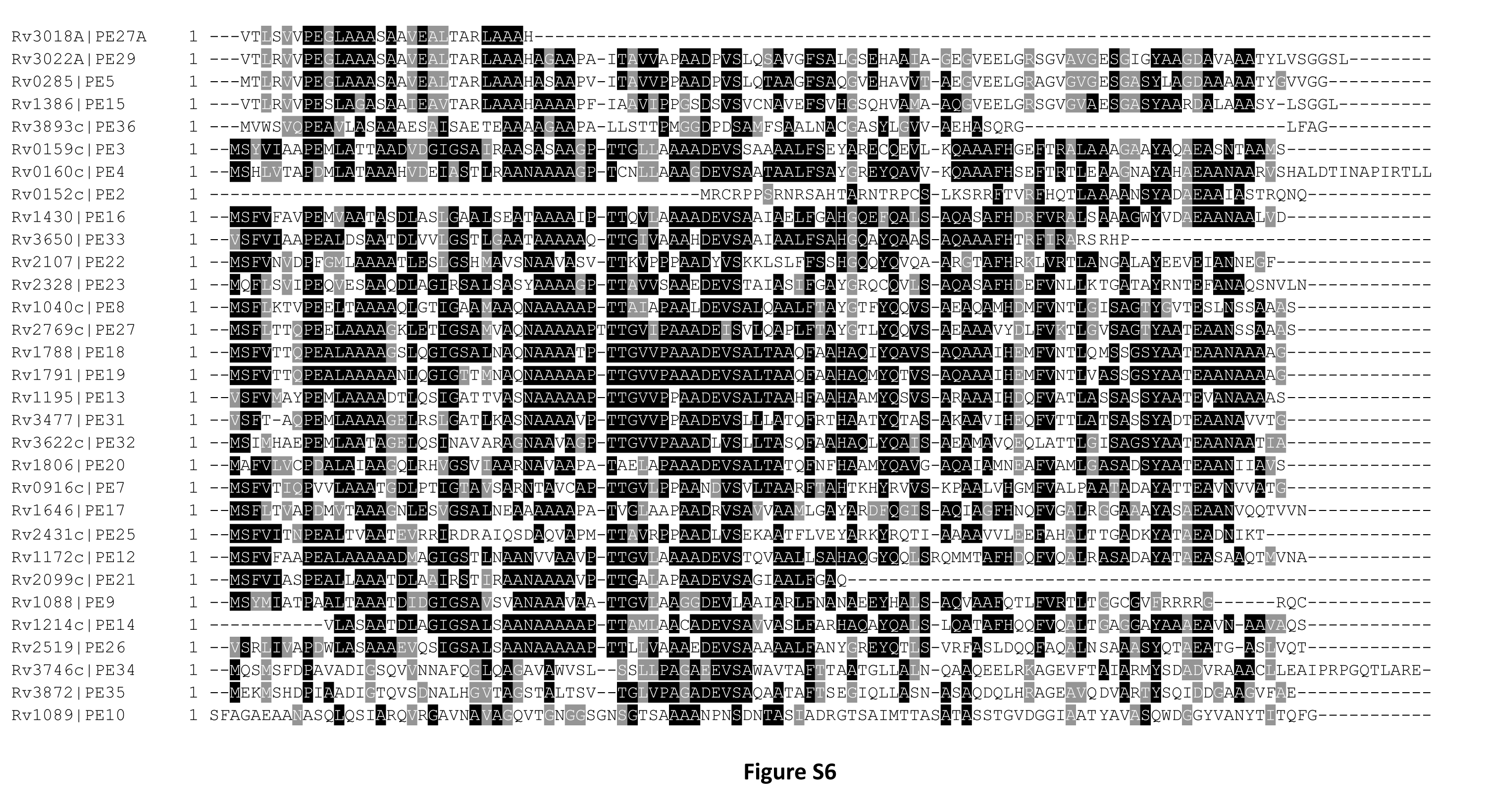

Supplement: Figure S6 — Sequence conservation among the PE subfamily of M.tb . Multiple sequence alignment of N-termini of 31/34 proteins of the ‘PE only’ sub-family; PE1, PE16 and PE24 were omitted from the alignment for illustrative purposes. (TIF) [file pone.0051686.s006.tif]
