# Supplementary material for: The Mycobacterium tuberculosis PE Proteins Rv0285 and Rv1386 Modulate Innate Immunity and Mediate Bacillary Survival in Macrophages
Source: PLoS One. 2012 Dec 17;7(12):e51686. doi: 10.1371/journal.pone.0051686 (PMC3524191; doi:10.1371/journal.pone.0051686)
Supplement: Table S1 — Oligonucleotides used in this study. (DOCX) [file pone.0051686.s007.docx]

**Table S1. Oligonucleotides used in this study**

| **Name** | **Comments** | **Sequence** |
| --- | --- | --- |
| pMV261- PE5-F | FP (Forward Primer) for cloning *PE5* in pMV261 | 5’ CAGTCAGATCTATGACGTTGCGAGTGGTTCCG 3’ |
| pMV261- PE5- R | RP (Reverse Primer) for cloning *PE5* in pMV261 | 5’ CATGCGAATTCTCAGCCGCCCACGACCCCG 3’ |
| pMV261-PE15-F | FP for cloning *PE15* in pMV261 | 5’ GTGTCGGATCCGTGACGTTGCGAGTCGTTCC 3’ |
| pMV261-PE15-R | RP for cloning *PE15* in pMV261 | 5’ CTCGGGAATTCTCATAGCCCACCGCTGAGATA 3’ |
| pJEX55-PE5-F | FP for cloning *PE5* in pJEX55 | 5’ CAGTCAGATCTATGACGTTGCGAGTGGTTCCG 3’ |
| pJEX55-PE5-R | RP for cloning *PE5* in pJEX55 | 5’ CCATG GAATTCGCCGCCCACGACCCCGTAC 3’ |
| pJEX55-PE15-F | FP for cloning *PE15* in pJEX55 | 5’ GAATTCTAGCCCACCGCTGAGATACGA 3’ |
| pJEX55-PE15-R | RP for cloning *PE15* in pJEX55 | 5’ TCAGTCCATATGACGTTGCGAGTGGTTCCGGAG 3’ |
| pET22b-PE5-F | FP for cloning *PE5*in pET22b | 5’ CCATGCCTCGAGGCCGCCCACGACCCCGTAC 3’ |
| pET22b-PE5-R | RP for cloning *PE5* in pET22b | 5’ GCGGCGCATATGGCCCCGTTTATCGCGGCGG 3’ |
| pET22b- NC- PE15F | FP for cloning *PE15* in pET22b | 5’ GCTCGGCTCGAGTAGCCCACCGCTGAGATACGA 3’ |
| pET22b- NC- PE15R | RP for cloning *PE15* in pET22b | 5’ ATGACGTTGCGAGTGGTTCCG 3’ |
| RT-PE5- F | FP for expression analysis of *PE5* in *M.smegmatis*(RT PCR) | 5’ GGCGGTGACGACCGCGTG 3’ |
| RT-PE5 -R | RP for expression analysis of *PE5* in *M.smegmatis* | 5’ GTGACGTTGCGAGTCGTTCC 3’ |
| RT-PE15- F | FP for expression analysis of *PE15* in *M.smegmatis* | 5’ TGAGCGGCCATTGCCACATG 3’ |
| RT- PE15- R | RP for expression analysis of *PE15* in *M.smegmatis* | 5’ ATGGCCGCGCCCATCTGG3’ |
| PPE4- F | FP for co-expression analysis of *PPE4* in *M.tb* | 5’ CCGCCTGATAAAGACCCATG 3’ |
| PPE4-R | RP for co-expression analysis of *PPE4* in *M.tb* | 5’ TTACTTGCTGTCGTGCGGTAAG 3’ |
| PPE4-R1 | Primer for first strand synthesis of the *PE5-PPE4* gene pair | 5’ GTTGGTGTGGGCGAATCCG 3’ |
| PE5- JF | FP for co-expression analysis of the *PE5-PPE4* junction in *M.tb* | 5’ GGCCAACTCCGCTAATGTC 3’ |
| PPE4-JR | RP for co-expression analysis of the *PE5-PPE4* junction in *M.tb* | 5’ GGCCAACTCCGCTAATGTC 3’ |
| PPE20-F | FP for co-expression analysis of *PPE20* in *M.tb* | 5’ATGACCGAGCCGTGGATAG 3’ |
| PPE20 -R | FP for co-expression analysis of *PPE20* in *M.tb* | 5’ GCGGAGAGCGCCGATCTG 3’ |
| PPE20J-R | FP for co-expression analysis of the *PE15-PPE20* junction in *M.tb* | 5’ CGCGAGCTTGATTTGGTTGG 3’ |
| PE15J -F | RP for co-expression analysis of the *PE15-PPE20* junction in *M.tb* | 5’ AGTTATGCCGCTAGGGATGC 3’ |
| PPE20-R1 | Primer for first strand synthesis of the *PE15-PPE20* gene pair | 5’ TCAGCGATGCTCGTCGAACA 3’ |
| huIL10 RT -F | FP for RT PCR of human IL-10 | 5' CCTTGTCTGAGATGATCCAGTT 3' |
| huIL10 RT -R | RP for RT PCR of human IL-10 | 5' TAAAGGCATTCTTCACCTGCTC 3' |
| huActin β RT-F | FP for RT PCR of human β-actin | 5' GAGCAAGAGAGGCATCCTCAC 3' |
| huActin β RT-R | RP for RT PCR of human β-actin | 5' CTCAAACATGATCTGGGTCATC 3' |
| huIL12b RT-F | FP for RT PCR of human IL-12b | 5' ATCAGGGACATCATCAAACCTG 3' |
| huIL12b RT-R | RP for RT PCR of human IL-12b | 5' AGGTCTTGTCCGTGAAGACTC 3' |
| huGAPDH RT-F | FP for RT PCR of human GAPDH | 5' AAGGACTCATGACCACAGTCC 3' |
| hu GAPDH RT-R | RP for RT PCR of human GAPDH | 5' GACACGGAAGGCCATGCCAG 3' |
| hu RT INOS-F | FP for RT PCR of human iNOS2 | 5’ AGTTTCCAGAAGCAGAATGTGAC 3’ |
| hu RT INOS-R | RP for RT PCR of human iNOS2 | 5’ GTAGAAAGGGGACAGGACGTA 3’ |
| Mm Actin β RT-F | FP for RT PCR of murine β-actin | 5' GATGACCCAGATCATGTTTGAG 3' |
| Mm Actin β RT-R | RP for RT PCR of murine β-actin | 5' CCAGCCAGGTCCAGACGCA 3' |
| Mm GAPDH RT-F | FP for RT PCR of murine GAPDH | 5' ACCTGCCAAGTATGATGACATC 3' |
| Mm GAPDH RT-R | FP for RT PCR of murine GAPDH | 5' ATTGTCATACCAGGAAATGAGC 3' |
| MmIL4 RT-F | FP for RT PCR of murine IL-4 | 5' CAGCAACGAAGAACACCACAG 3' |
| MmIL4 RT-R | RP for RT PCR of murine IL-4 | 5' GACTTGGACTCATTCATGGTG 3' |
| MmIL12b RT-F | FP for RT PCR of murine IL-12b | 5' GGACATCATCAAACCAGACC 3' |
| MmIL12b RT-R | RP for RT PCR of murine IL-12b | 5' CGCACCTTTCTGGTTACACC 3' |
| MmIL5 RT-F | FP for RT PCR of murine IL-5 | 5' TGACAAGCAATGAGACGATGAG 3' |
| MmIL5 RT-R | RP for RT PCR of murine IL-5 | 5' CTCTCCTCGCCACACTTCTC 3' |
| MmTGF-β RT-F | FP for RT PCR of murine TGF-β | 5’GACAGCAAAGATAACAAACTCCA 3’ |
| MmTGF-β RT-R | RP for RT PCR of murine TGF-β | 5’CTTCTCTGTGGAGCTGAAGCA3’ |
| MmINOS-F | FP for RT PCR of murine INOS2 | 5’ TTCAGGACATCCTGCAAAAGC 3’ |
| MmINOS-R | RP for RT PCR of murine INOS2 | 5’ TCTGGCTCTTGAGCTGGAAG 3’ |
| *MssigA* RT-F | FP for RT PCR of *M.smegmatis* sigA | 5’GCCAGCTCGGTGACTTCA3’ |
| *MssigA* RT-R | RP for RT PCR  *of M.smegmatis* sigA | 5’CGTGACGCCGTAGACCTG3’ |
| pJEX55NCMPT64 F | FP for cloning NCMPT64 in pJEX55 | 5’TTACGGATCCATGGCGCCCAAGACCTACTGC 3’ |
| pJEX55NCMPT64 R | RP for cloning NCMPT64 in pJEX55 | 5’GCGAGT GAATTC GGCCAGCATCGAGTCGATC 3’ |
| pJEX5MPT64 F | FP for cloning MPT64 in pJEX55 | 5’TTCGACGGATCCGTGCGCATCAAGATCTTCATG3’ |
| pJEX5MPT64 R | RP for cloning MPT64 in pJEX55 | 5’GCGAGT GAATTC GGCCAGCATCGAGTCGATC 3’ |
| pJEX55PE11 F | FP for cloning PE11 in pJEX55 | 5’CACGCGGATCCGTGTCTTTTGTCACCACACGG3’ |
| pJEX55PE11 R | FP for cloning PE11 in pJEX55 | 5’ GCTGTGAATTCGGTGGAGGTGCCCGCGCG 3’ |
| pJEX55Nter-HtrAF | FP for cloning Nter-HtrA in NCMPT64 | 5’ATTAGGATCC GTGGATACTAGG GTGGACAC3’ |
| pJEX55Nter-HtrAR | FP for cloning Nter-HtrA in NCMPT64FP f | 5’ ATTT GAATTCGGTCACCACCGAATCGGC 3’ |
